# Global Optimization of Molybdenum Subnanoclusters on Graphene: A Consistent Approach toward Catalytic Applications

**DOI:** 10.1021/acsami.4c13102

**Published:** 2024-11-06

**Authors:** Yao Wei, Alejandro Santana-Bonilla, Lev Kantorovich

**Affiliations:** Theory and Simulation of Condensed Matter (TSCM), King’s College London, Strand, London WC2R 2LS, U.K.

**Keywords:** global optimization, particle swarm optimization, graphene, molybdenum, adsorption, catalysis

## Abstract

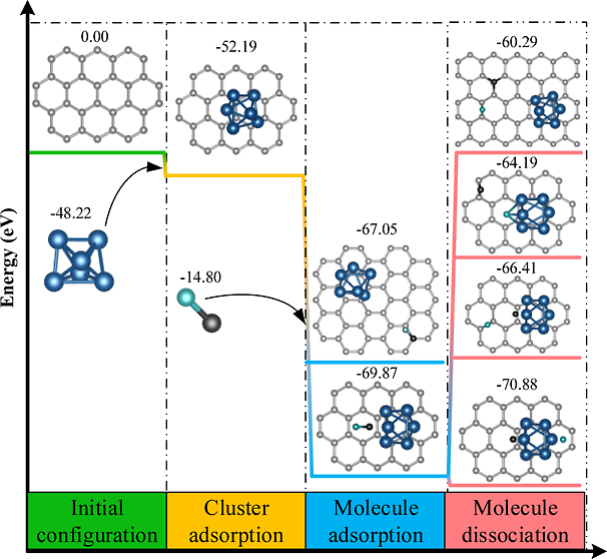

The development of novel subnanometer clusters (SNCs) catalysts with superior catalytic performance depends on the precise control of clusters’ atomistic sizes, shapes, and accurate deposition onto surfaces. The intrinsic complexity of the adsorption process complicates the ability to achieve an atomistic understanding of the most relevant structure–reactivity relationships hampering the rational design of novel catalytic materials. In most cases, existing computational approaches rely on just a few structures to draw conclusions on clusters’ reactivity thereby neglecting the complexity of the existing energy landscapes thus leading to insufficient sampling and, most likely, unreliable predictions. Moreover, modeling of the actual experimental procedure that is responsible for the deposition of SNCs on surfaces is often not done even though in some cases this procedure may enhance the significance of certain (e.g., metastable) adsorption geometries. This study proposes a novel systematic approach that utilizes global search techniques, specifically, the particle swarm optimization (PSO) method, in conjunction with ab initio calculations, to simulate all stages in the beam experiments, from predicting the most relevant SNCs structures in the beam and on a surface, to their reactivity. To illustrate the main steps of our approach, we consider the deposition of Molybdenum SNC of 6 Mo atoms on a free-standing graphene surface, as well as their catalytic properties with respect to the CO molecule dissociation reaction. Even though our calculations are not exhaustive and serve only to produce an illustration of the method, they are still able to provide insight into the complicated energy landscape of Mo SNCs on graphene demonstrating the catalytic activity of Mo SNCs and the importance of performing statistical sampling of available configurations. This study establishes a reliable procedure for performing theoretical rational design predictions.

## Introduction

Transition-metal subnanometer clusters (SNCs) have emerged as promising novel catalytic materials, demonstrating superior performance compared to their metallic and transition metal nanoparticle counterparts.^1^ This remarkable enhancement arises from their high surface-to-volume ratio, which facilitates a unique catalytic behavior due to a combination of low atomic coordination number and electronic accessibility to transition-metal atoms to reactants.^2^ Advancements in synthesis and characterization techniques have enabled the production of atomically precise transition-metal SNCs, with the ultimate experimental objective being the development of scalable and cost-effective methods to synthesize these materials with tailored properties.^3^ Therefore, developing a rational design strategy is paramount to effectively deploy these materials in a diverse range of applications, such as energy storage, photocatalysis, and biomedicine.^4−6^

Despite this experimental progress, transition-metal SNCs are likely to sinter and lose surface area when used as catalysts.^7^ To prevent this, supported solid-state materials (such as carbon or metal oxide surfaces) are used to stabilize transition-metal SNCs.^8,9^ Among many experimental techniques, the beam deposition methods^10^ have been successfully tested for depositing on different substrates atomically precise transition metal (TM) clusters with a predefined number of atoms. In these methods, an induced positive charge of the clusters enables one to direct them precisely onto the surface, while their low kinetic energies prevent the clusters from decomposing when landing on it (the so-called “soft landing”). Moreover, it is assumed that upon contact the clusters instantaneously get the electronic charge from the surface and end up adsorbed in the neutral state.

Due to the complexity of the atomic-level process described, developing a theoretical understanding of the structure–reactivity relationship for the clusters on the surface is crucial.^11^ From an atomistic perspective, exploring the accessible thermodynamic states of the clusters on the surface is challenging due to the huge variety of possible clusters and numerous degrees of freedom that must be considered. Fortunately, the atomic-scale control offered in the beam experiments^12^ (whereby the number of atoms in the clusters is known) reduces enormously the amount of theoretical work required to forecast clusters’ adsorption geometries on the surface of interest, calculate their electronic structure, including their magnetic states, and consequently predict their reactivity (catalytic activity) toward specific chemical reactions. Note that, from the theoretical point of view, the problem would otherwise be unmanageable due to the extreme complexity of the energy landscape and hence the necessity of considering an enormous number of possible adsorption systems with a wide range of adsorption energies.

Even due to the mentioned significant reduction of possibilities, one still needs to find the most probable adsorption geometries to be realized in the beam experiments, some of which (or all) could be metastable. Straightforward geometry optimization strategies relying on guessing initial geometries are still abundant in the literature, for recent examples see, e.g., refs (13–19) However, these naive approaches are most likely inapplicable here. Modern computational procedures, such as meta-heuristic algorithms including genetic algorithms,^20−26^ random sampling,^27−29^ data mining,^30^ simulated annealing,^31,32^ basin hopping,^33^ and Particle Swarm Optimization (PSO),^34^ can provide a more effective search process by employing efficient exploration techniques of the configurational space associated with a specific optimization problem. Mentioned above and some other global optimization techniques are reviewed in refs (35–37) Moreover, an additional advantage of most of these methods is that they also offer a range of metastable geometries with energies near the global minimum, which may even be more reactive.^37^

In this work, we employ global minimum search methods, such as the ab initio random structure searching (AIRSS)^27,29^ and PSO algorithm^38,39^ to investigate the energy landscape of a series of Molybdenum SNCs adsorbed onto pristine free-standing graphene in the beam experiments. Note that the PSO algorithm was highly rated in a recent study^36^ for its stable performance compared to other methods. The adsorption of transition-metal SNCs onto an inorganic support profoundly influences their properties, including geometry, electronic structure, and charge state, ultimately dictating the catalytic performance of the system. We have chosen Molybdenum as it is well-known for its catalytic properties.^40−42^ Using a unified approach based on stochastic methods, we study the entire experimental procedure, starting from the clusters in the beam and their adsorption on the surface, and then moving on to investigate their reactivity with respect to the dissociation of a CO molecule. The results of these studies indicate that the predicted Molybdenum SNCs do indeed exhibit catalytic activity.

Literature on combined Molybdenum and CO molecule systems is rather scarce. There have been some experimental and computational studies on the interaction of the CO with Mo(100), Mo(110) and Mo(112) surfaces, in which dissociation of the CO has also been discussed.^43−49^ We are also aware of a single study of the chemical reactivity of Mo_*n*_ clusters (with *n* ≤ 14) toward binding the CO molecule,^50^ in which, however, dissociation of the latter was not considered. In the computational study^51^ CO dissociation on the Mo_3_ gas phase cluster was investigated where a few eV energy barrier was obtained. To the best of our knowledge, there have been no studies of reactivity of Mo clusters adsorbed on graphene or graphite surfaces toward the CO dissociation reaction, neither experimental nor computational.

If each particular step of the experimental procedure has been simulated and reported previously, e.g., gas-phase clusters or clusters’ adsorption on a surface, we are not aware of a consistent and systematic global optimization approach applied throughout all steps of the whole procedure. To the best of our knowledge, this is the first study of that kind that simulates computationally the entire experimental protocol, from the formation of the clusters in the beam and their adsorption on a surface to a chemical reaction catalyzed by them.

We stress that the main purpose of this work is to emphasize the importance of thoroughly exploring the potential energy landscape in investigating every step of the experimental procedure, and that, due to overall complexity of the problem, stochastic methods must be used at each stage to properly sample the corresponding configurational phase space. Following this paradigm in full would require substantial computational resources. Therefore, in this work, as the first step toward the full approach, we have just tried to illustrate the method by selecting a simple system (Mo clusters of 6 atoms and the free-standing graphene as a surface) and follow at each step just a subset of available opportunities. We have found this procedure sufficient to understand the multitude of various possibilities one has to tackle when studying these kinds of problems, and appreciate the necessity of thoroughly exploring the potential energy surface. A fully comprehensive study is left for future work.

## Methods

### General Computational Strategy and Computational Details

**Figure 1:**
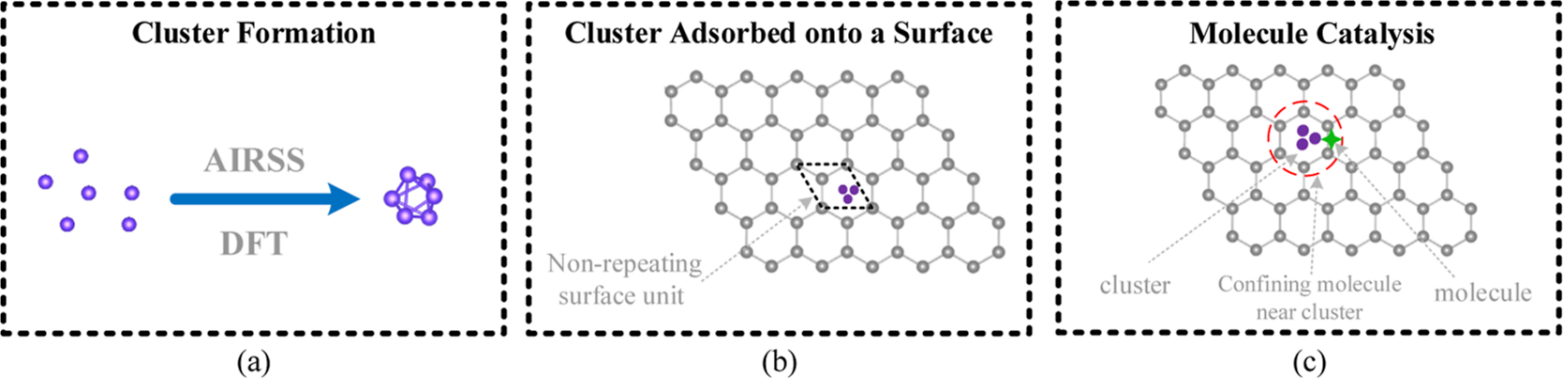
Schematic Representation of the simulation approach followed in this study. (a) Positively charged clusters in the gas phase. (b) Clusters adsorbed onto a surface. (c) Consideration of the catalysis at and around the cluster.

**Figure 2:**
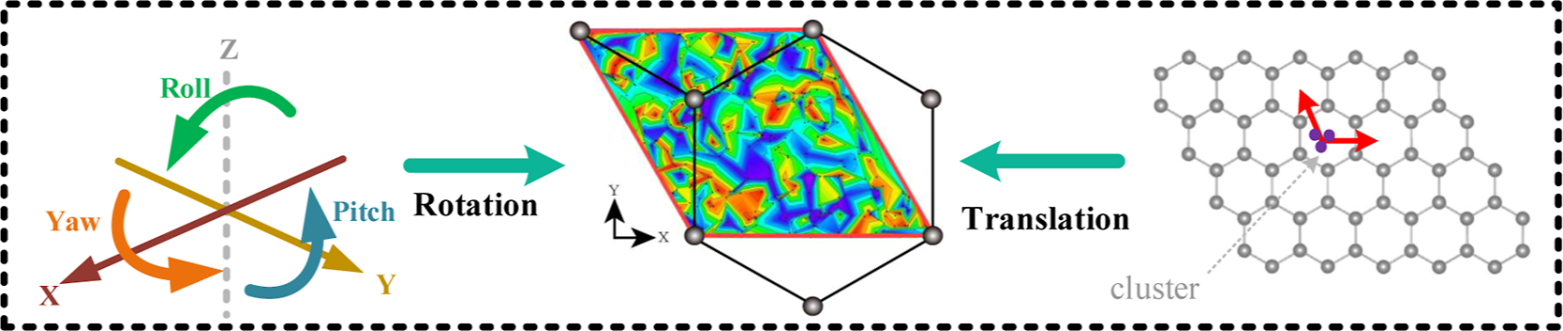
Schematic representation of a 5D multiobjective PSO framework for surface-cluster adsorption. This scheme considers 3 rotations and 2 translations, yielding projected potential energy surface as a function of cluster exploration.

We propose the following general computational strategy that can be split into several interconnected stages:

1. In the beam experiments^12,52^ monatomic clusters are ejected by an atomic gun and ionized. Carrying a single positive charge, it is then possible to affect the direction they move in, select the clusters of a particular mass and, therefore, of the desired number of atoms, Figure 1a. This means that one can experimentally select the monatomic clusters of a predefined number of atoms in the beam and direct them onto the surface of interest with great precision. Moreover, because of the clusters’ charge, it is possible to control their kinetic energy, which enables one to land the clusters “softly” on the surface. From theoretical point of view, this means that one can use their equilibrium gas-phase geometry as the initial structure, prior to geometry relaxation due to the interaction with the surface. The soft landing of Au clusters on the TiO_2_ surface was, e.g., modeled using molecular dynamics simulations in ref (53) It is well-known that the final geometry in the geometry relaxation process in many cases is influenced by the initial geometry, i.e. instead of the global energy minimum, metastable structures would normally be established. Hence, to find clusters’ geometries on the surface, one needs first to find the lowest energy structures of positively charged clusters in the gas phase. Once these are known, we would be able to consider mostly the cluster(s) of lowest energy(ies) in placing them on the surface, prior to geometry relaxation. So, at the first stage of our computational approach, we are interested in investigating possibly all lowest energy clusters of a single positive charge in the gas phase.
2. At the next stage, we intend to put these clusters on the surface of interest. However, this can be done in many ways as clusters would explore the free-energy landscape generated by the surface, seeking the most stable configuration for the attachment, as illustrated in Figure 2. However, the most appropriate way of choosing the clusters’ adsorption sites is instigated by the experimental procedure itself. Indeed, the clusters in the beam may land on the surface at different lateral positions (via a uniform distribution in a uniform beam) and at different spatial orientations (again, with equal probability). Therefore, we have designed a procedure (to be detailed below) whereby the lowest gas-phase energy clusters are placed on the surface at random, at various lateral positions (within the periodic cell) and various orientations in space. There are five variables here to vary: two lateral positions and their angles that uniquely specify the clusters’ orientation. It is assumed that upon landing the clusters will almost immediately (at electronic time scales) get neutralized on the surface via an electron transfer from it;^54,55^ hence, we can proceed with the subsequent geometry relaxation of the clusters on the surface considering the whole system is neutral. After accomplishing this stage, we shall have the lowest energy structures of the clusters on the surface that are attainable in this type of experiment. It is essential to realize two points here: (i) most likely, there will be many geometries of the clusters with energies close to the lowest energy configuration, and hence all of them need to be taken into consideration while studying applications, e.g., in catalysis, and (ii) the obtained geometries of the clusters with *n* atoms will most likely not correspond to the global energy minimum of the surface with *n* individual atoms, as the obtained final geometries will be influenced by the clusters’ initial geometries in the beam prior to their contact with the surface. It is believed that the obtained geometries of the clusters on the surface would correspond to the majority of such clusters in the considered experimental procedure. Hence, finding the lowest energy structures of the clusters in the gas phase and then exploring their adsorption comprises two interlinked essential parts of our computational strategy that is meant to model this particular beam experiment.
3. At the third stage we shall study the role of these clusters in catalyzing a dissociation chemical reaction. To this end, we need first to find all lowest energy adsorption sites of the molecule of interest at or near one of the clusters found, to be considered as the initial geometries of the reaction (reactants), Figure 1c. Next, in a similar fashion, all possible lowest energy structures of the products of the reaction at or near the cluster are to be determined using a similar procedure, to be considered as the final geometries after the reaction. Then, all the initial and final structures need to be connected by minimum energy path calculations to determine the corresponding energy barriers and hence the transition rates. These calculations are to be repeated for all lowest energy clusters on the surface found.
4. The paths corresponding to the lowest energy barriers, in conjunction with the lowest energy clusters (that determine their abundance on the surface) are to be considered as defining the chemical reaction of interest. Hence, at the last stage, we consider kinetics of the dissociation process in which all clusters participate and all obtained dissociation paths are included explicitly. The initial concentrations (or populations) of the various clusters prior to the reaction (upon deposition) are determined by the canonical statistical distribution based on their formation energies, and the evolution in time of the products—by the obtained transition rates based on the calculated energy barriers. Since it is assumed that during the course of the reaction diffusion of reactants and their possible transformations are unlikely (which as well may not be true), the rate equation approach^56−58^ will be employed to study the time dynamics of the dissociation reaction. If the surface diffusion and structural fluxionality effects^18,37,59^ are thought to play an essential role, methods such as kinetic Monte Carlo (kMC)^60,61^ will have to be employed (see, also refs (58,59,62)).

To accomplish each stage of the above procedure, we shall employ stochastic approaches that are the most suitable in each case as detailed in the next subsection.

### Computational Methods

To obtain the total energies of the systems, we have used ab initio computational method based on the Density Functional Theory (DFT) approach. All DFT calculations have been performed using the VASP (Vienna Ab initio Simulation Package) code^63,64^ using the Perdew, Burke, and Ernzerhof (PBE) generalized gradient approximation (GGA) functional.^65^ Additionally, the van der Waals (vdW) dispersion correction was introduced by employing Grimme’s D3 method.^66^ Electron–ion interactions were described using the projector-augmented wave (PAW) method^67^ with electronic configurations 2s^2^2p^2^ and 4s^2^4p^6^4d^5^5s^1^ for C and Mo atoms, respectively. The Mo configuration chosen was found necessary in our previous study of gas-phase Mo clusters.^68^ The calculations were performed using several values of the number of unpaired electrons in the system to account for the possibility of different spin states. Due to the large sizes of the periodic cells used (see below), the Γ-point has been found sufficient to sample the Brillouin zone in all our calculations. A plane-wave kinetic-energy cutoff of 400 eV was used, and each relaxation the calculation was considered finished when the energy threshold of 10^–5^ eV was reached, while the atomic forces were not larger than 0.01 eV/Å. To perform DFT simulations on charged Mo clusters, we have used a standard procedure implemented in VASP in which a neutralizing background is implemented and a Coulomb correction is applied to weaken the inevitable dependence on the cell size. When performing adsorption calculations, we chose a simulation cell containing 72 carbon atoms that is obtained by expanding the primitive cell of graphene (2 atoms in the primitive cell) along two primitive lattice vectors by 5 and 6 times, respectively. To prevent the translation of the whole system during the relaxation, a few atoms of graphene that are remote to the cluster were kept fixed. Spin polarization is considered in our calculations, employing an initial magnetic moment of 1.0 Bohr magnetons (μ_B_) for each Mo atom.

To compute the minimum energy path in the CO dissociation reaction between the initial position of the CO molecule near or at a Mo cluster and the final positions of the individual C and O atoms after the reaction, we used the Nudged Elastic Band (NEB) method^69^ as implemented in the CINEB + VASP code.^70^ An appropriate number of images (as indicated below) was used in all NEB simulations and each calculation was considered converged when the energy and forces were better than 10^–5^ eV and 30 meV/Å, respectively.

### Algorithms’ Implementation

At the first stage, to simulate the process of cluster formation in the gas phase, we employed the AIRSS (Ab initio Random Structure Searching) random structure search package^29^ to identify the lowest energy gas-phase structures. AIRSS achieves this by considering, at random, various physical features, such as atomic distances and symmetry. The software systematically explores the configurational space to identify promising atomic structures and has been successfully applied by some of us previously to study neutral Molybdenum SNCs.^68^ Using this approach we were not only able to find Mo clusters proposed previously, but also to predict many new ones, of very different geometry and electronic and magnetic structure. The AIRSS approach is well suited for considering low-energy structures formed by a set of individual atoms as it enables one to construct many possible compositions.

To navigate the intricate configurational landscape created by clusters on the surface, AIRSS is no longer appropriate as we need to consider at random only the lateral position of the whole cluster of known geometry and its orientation, 5 degrees of freedom in total upon its adsorption on the surface. Hence, we have chosen the Particle Swarm Optimization (PSO) technique as a promising approach to effectively pursue the vast array of possibilities arising from this process. PSO is a population-based stochastic optimization method inspired by the social interactions observed in bird flocks and fish schools. In PSO, each particle represents a potential solution to the optimization problem, with its position corresponding to a candidate solution and its velocity governing its movement through the configurational space. PSO has gained widespread use across various domains, including function optimization, parameter tuning, data clustering, and crystal structure prediction.^34^ Its simplicity, computational efficiency, and ability to tackle multimodal and nonlinear problems make it a popular choice for optimization tasks. We stress that it is not guaranteed that the best structure found by PSO is the true global energy minimum; however, with each generation (iteration), as the configurational space is increasingly better explored, the best structures obtained become lower and lower in their energies, thereby (at least) consistently approaching the global energy minimum. Note that the method offers a possibility of keeping lower energy structures that are found during the course of the optimization as well, thereby providing, at no additional cost, a sample of many energetically favorable structures.

In the PSO framework, each potential solution (in our case, this comprises a 5-fold vector **X** of the lateral position and orientation of a cluster) is represented by a particle, and a collection of these particles at every given iteration forms a generation. These particles navigate the search space, influenced by their own best-known positions and the best positions of their neighbors. Initially, a swarm of particles with random positions and velocities is established within the search space. Subsequently, the fitness of each particle is evaluated, reflecting the quality of the solution it represents. In our case the fitness is determined by the DFT energy of the structure obtained after geometry relaxation from the initial geometry given by the vector **X**. This was found essential as otherwise (if the PSO was run without geometry optimization) some of the structures that upon optimization have low energies may be rejected as they appear at the end of the list. In performing the geometry relaxation, the vertical position of each cluster prior to the relaxation was in all cases chosen as 3.0 Å between the graphene and the lowest cluster atom. The rationale for choosing this particular initial vertical position is to ensure that, on the one hand, the cluster will not crash onto the surface, and, on another, it is not too far from it either so that there is a numerically significant force to pull it down. The personal best position **X**_best_ for each particle is updated, representing the particle’s most promising position explored so far. Next, the particle with the best **X**_best_ across the whole swarm of particles of the current generation is identified, representing the current best solution found by the swarm. This particle’s position is termed the global best position **X**_g-best_. For a new generation *t* + 1, the “velocity” of *i*-th particle is updated based on its current position **X**_*i*_(*t*) in the current generation *t* as well as the values of **X**_best_ and **X**_g-best_ following the formula
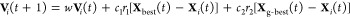
1where ω represents the inertia weight, while *r*_1_ and *r*_2_ are uniformly distributed random numbers *U*(0, 1). The constant *c*_1_ determines the influence of a particle’s own experience on its movement (Personal Learning Rate), whereas *c*_2_ determines the influence of the swarm’s collective knowledge (Social Learning Rate). Finally, the position of the particle is updated via
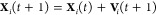
2producing the particle’s new location in the search space (a new generation). For this work, we have used 0.1, 0.16, and 0.84 for ω, *c*_1_, and *c*_2_, respectively. These values for the parameters were determined through a series of benchmark tests aimed at optimizing the balance between exploration and exploitation within our specific model context. We used a swarm of 20 particles (i.e different initial cluster configurations). As was mentioned before, at each step the position **X**_*i*_(*t*) of the particle is used to work out the initial geometry of the cluster on the surface prior to the DFT geometry optimization (see the Supporting Information file) in which both the graphene surface and the cluster atoms were allowed to relax (apart from a few fixed graphene atoms, as mentioned earlier); upon relaxation, the final DFT energy represents the fitness of the particle in the swarm that determines its position in it and hence which of the structures in the swarm is the best.

As was explained above, during the search for the optimal adsorption configuration, only the lateral position, given by the center of mass, and the orientation of the clusters, given by three rotational angles (two translational coordinates and three angles), are employed in the PSO algorithm (see the Supporting Information file for details). The sensitive nature of these five control parameters during the cluster displacement and rotation makes it challenging to avoid unfavorable local minima in the adsorption site search. To mitigate this issue, approximately 40% of the least favorable structures were discarded in each generation, and their corresponding structural parameters were randomly regenerated (so that each generation always contained exactly 20 structures). The convergence of the generations in finding the adsorption site with the lowest overall energy depends on the complexity of the system; in current simulations, if the energy difference of the best ten systems in the simulation is smaller than 0.01 eV, we considered it as converged. Depending on the system, between 15 and 40 generations were required to reach this convergence criterion.

A modified version of the PSO method has also been used in predicting positions of the CO molecule prior to the dissociation reaction, as well as in predicting the position of the O atom after the reaction. To simplify these latter calculations, we have considered a single position of the C atom on the cluster only. Of course, a comprehensive approach, as was outlined in the previous subsection, would go through all possible positions of both C and O atoms as the final states of the reaction. That would undoubtedly increase the cost of these simulations, let alone the number of possibilities to consider in rather expensive NEB simulations that will follow. As our main aim in this work is mainly to highlight the complexity of the problem at hand and to indicate the appropriate workflow to tackle it, we find our limited approach outlined above to be sufficient here for our purposes.

As the number of DFT geometry relaxations during the course of the PSO search is counted into thousands and the size of the systems was much larger than for the gas-phase clusters within the AIRSS approach, larger tolerances of 5 × 10^–5^ eV (energy) and 50 meV/Å (forces) were used in all PSO simulations.

When using both AIRSS and PSO methods, hundreds of structures were generated that span a wide range of energies (see below). We found that in each case there are the significant number of structures generated with their energies very close to the best energy we find. A detailed analysis showed that many of the structures we find are essentially identical, hence, a necessity arose when using these stochastic approaches to automatically identify the identical structures and then discard them. Our method is close in spirit to the one considered in ref (71).

When performing AIRSS simulations on isolated clusters, we identified identical geometries by considering their symmetries. In PSO simulations for clusters on graphene, we encountered a complete lack of symmetry in the relaxed clusters. As a result, the identification of distinct adsorbed configurations became challenging through conventional symmetry analysis. To address this issue, we adopted an alternative approach by examining the spatial distribution of atoms within the cluster relative to its center of mass. Subsequently, we first organized the configurations based on their energy in the ascending order. If the spatial distributions were found to be closely aligned, indicating a similarity, we classified them as duplicates and excluded from further consideration. This methodology allowed us to discern and catalogue identical geometries within the PSO simulations.

## Results

### Lowest Energy Mo Clusters

In our recent work,^68^ we conducted an analysis of neutral Mo clusters comprising 3 to 10 atoms. That method was extended here for the investigation of Mo clusters with a single positive charge. To illustrate our approach, only clusters Mo_6_ containing 6 Mo atoms are considered.

**Figure 3:**
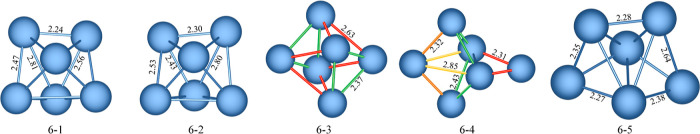
Lowest energy configurations of charged 6-atom Mo clusters. Mo–Mo bonds of the same length in clusters 6–3 and 6–4 that possess symmetry are shown in the same color for convenience.

Using the AIRSS method, we found more than 70 stable clusters with multiplicities equal to 2, 4, and 6. The total energies of the clusters span a huge energy range of over 10 eV from −43.24 to −32.95 eV. It is noted that all geometries within this energy range exhibit stability. However, within the energy range of 1.1 eV from the lowest energy cluster, only five clusters were found. These five lowest energy clusters are depicted in Figure 3 and their properties are shown in Table 1.

**Table 1:** Energies and Point Group Symmetries of the Charged Mo_6_^+^ Clusters

	6–1	6–2	6–3	6–4	6–5
total energy (eV)	–43.24	–42.61	–42.44	–42.30	–42.15
multiplicity	2	4	2	2	2
symmetry	*C*_1_	*C*_1_	*D*_3*d*_	*C*_*s*_	*C*_1_

When contrasting these results with the outcomes observed for neutral Mo_6_ clusters, we notice that the introduction of a single positive charge induces a reduction in the symmetry of the cluster as the lowest energy configuration exhibits *C*_1_ symmetry, whereas the neutral Mo_6_ lowest energy cluster shows *C*_2*v*_ symmetry. It is noteworthy that the cluster with the lowest energy is 0.6 eV lower in energy compared to the cluster with the second lowest energy. Note that the lowest energy cluster with multiplicity 6 (not shown) is nearly 1.2 eV higher in energy than the lowest energy cluster. It is clear that, because of the considerable energy difference, for this particular Mo_6_ system, it would be sufficient to proceed only with the single cluster of the lowest energy when placing the cluster on graphene and performing CO dissociation simulations, as described in the forthcoming subsections, as the populations of the other clusters in the gas phase (and correspondingly on the surface) will be statistically insignificant.

### Cluster Adsorbed onto the Free-Standing Graphene

Next, we selected the lowest energy gas-phase cluster 6–1 from Figure 3 and applied the PSO algorithm to find the lowest energy structures this cluster can form when adsorbed onto the graphene, when initially placed at different lateral positions and orientations, prior to geometry relaxation.

Prior to this calculation (see the Supporting Information file), we performed a PSO-based exploration of the best neutral cluster of 6 Mo atoms placed on graphene (using as its initial geometry the one obtained in our previous work^68^), and within 1.0 eV from the best structure we found altogether 9 lowest energy configurations with odd multiplicities between 1 and 7 (our system has an even number of electrons). Note that altogether, considering all geometries tried by the PSO approach, more than 90 structures were relaxed with the spread of energies of 2.24 eV. It was observed, however, that the first five best structures have multiplicities 1 and 3 only, with the sixth structure of multiplicity 5 being by 0.4 eV higher in energy than the best structure (of multiplicity 1). Based on these preliminary calculations, when placing the best 6–1 cluster on graphene (obtained in the gas phase assuming a single positive charge, see the previous Section), we limited ourselves to multiplicities 1 and 3 only. Note that the geometry of the isolated charged cluster was used in the PSO run only when placing the cluster on graphene in preparing its initial geometry prior to the DFT geometry relaxation; the latter was performed for the neutral system assuming an immediate electron transfer from graphene, as noted previously.

**Figure 4:**
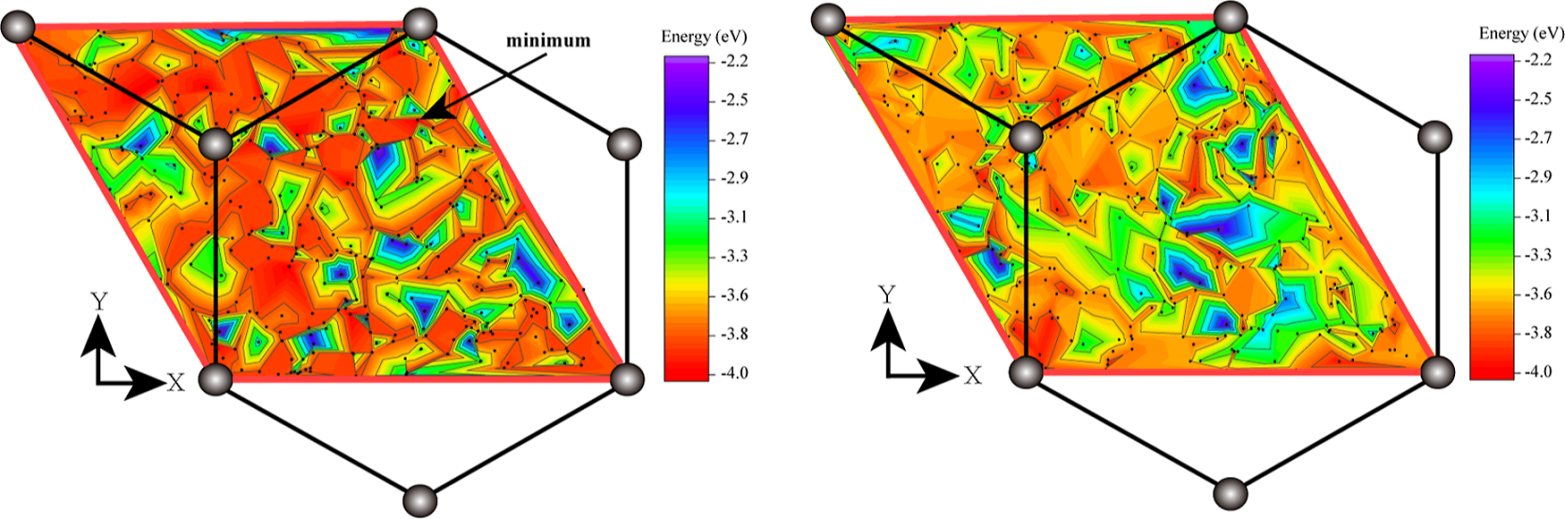
Map of the adsorption energy of the Mo_6_ cluster 6–1 adsorbed on graphene as a function of its lateral position for multiplicities *M* being 1 (left) and 3 (right). The graphene structure (shown by connected gray circles) is superimposed on the picture for convenience.

When performing the PSO-based exploration of the potential energy surface of our best Mo_6_ cluster 6–1 on graphene (using the geometry of the charged cluster as the initial one), 25 structures were tried overall with a total spread of 1.8 eV in their energies. In Figure 4 the distribution of these clusters over their energies are shown as a function of the clusters’ lateral position. Note that the three angles specifying the clusters’ orientation is ignored, i.e. each point on the potential adsorption energy surface (PES) (which is basically a projection of the whole 5-dimensional PES onto the cluster’s 2D lateral position subspace) corresponds to some values of the angles that are most likely to be different from point to point.

**Figure 5:**
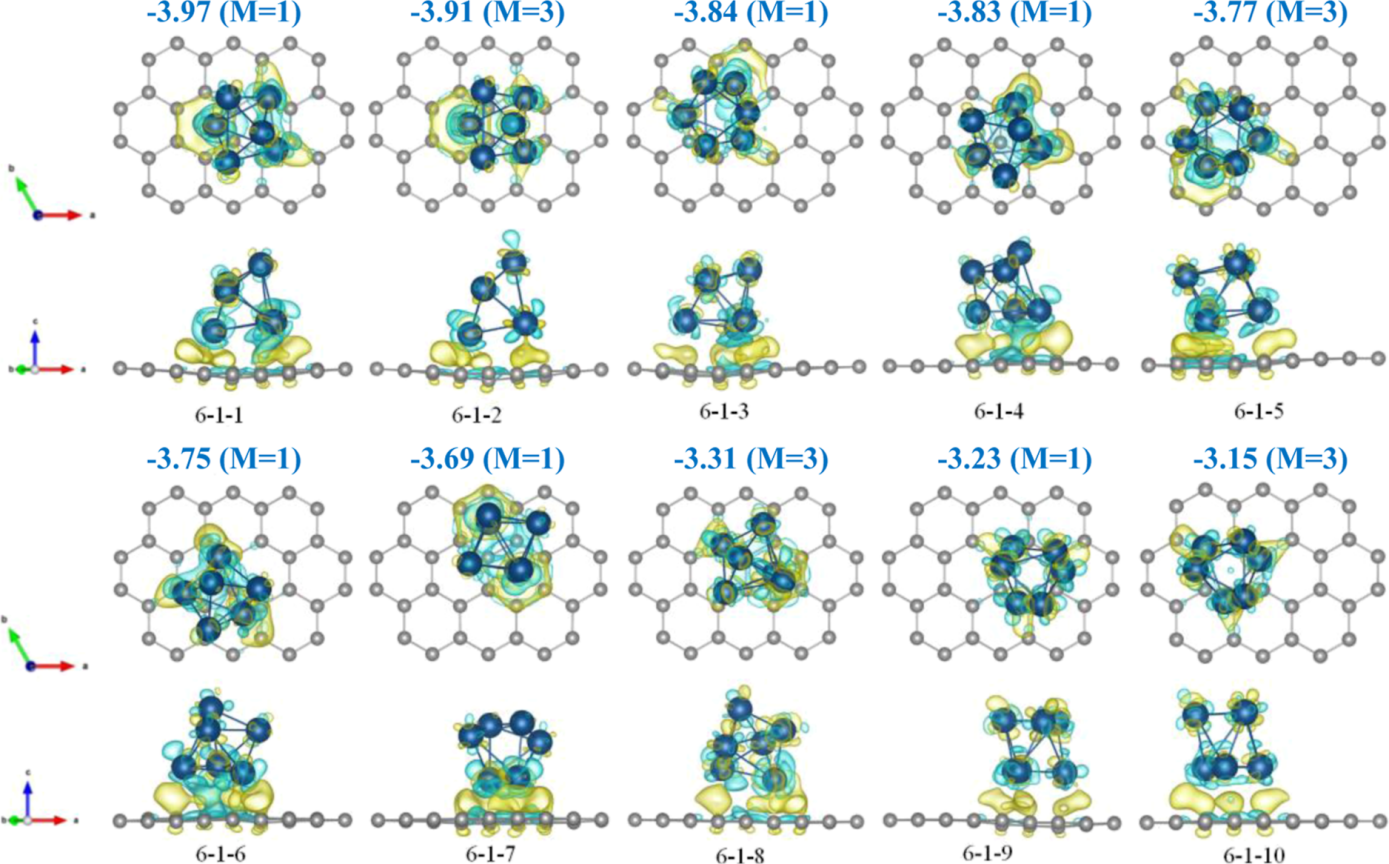
Top (above) and side (below) views of the best ten geometries of the Mo_6_ cluster adsorbed on graphene using the best 6–1 charged cluster geometry as the initial guess. The adsorption energies (in eV) and multiplicities *M* of the clusters (in μ_B_) are also shown. In each case the charge density difference is also shown. The green and yellow colors correspond to depletion and excess of the density (at ±0.005 Bohr^–3^), respectively.

The substantial variation of the PES and its complexity show, in particular, how important it is to explore the PES in cases of complicated systems in order to approach as close as possible the global minimum and hence avoid nonrepresentative results that may mislead further study and the conclusions to be made. The ten lowest energy structures obtained within 1 eV energy range are shown in Figure 5 together with their adsorption energies and multiplicities *M*. As usual, the adsorption energy is defined as the difference
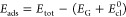
of the total energy *E*_tot_ of the combined system (the graphene and the cluster) and of the individually relaxed graphene *E*_G_ and the neutral cluster *E*_cl_^0^; the latter energy was obtained by considering the geometry of the relaxed charged cluster 6–1 in the neutral charge state. It is seen that the differences in the adsorption energies among the best four structures are relatively small, within 0.14 eV.

It is interesting to observe that for the particular system we are considering here, the four lowest-energy structures obtained in the PSO calculation are the same when using, as the initial geometry, either the charged or neutral clusters (small energy differences and hence a different order in Figures 5 and S1 in Supporting Information is explained by slightly different tolerances used in the two simulations). Note, however, that for other systems this may not be the case.

In Figure 5 we also show the density difference for selected systems. The density difference is defined in a similar way to the definition of the adsorption energy, by subtracting from the density of the whole system the densities of the two fragments (the graphene and the cluster, the latter is considered as neutral); note, however, that the geometries of the fragments correspond to their geometry in the combined system, i.e. not individually relaxed. It is seen that in all cases there is some charge transfer between the clusters and graphene, as well as some redistribution of the density within the clusters and in graphene just underneath.

As all our calculations were spin-polarized, we have access to the spin densities of our systems. Only systems with multiplicity 3 demonstrated a nonzero spin density, as expected, see Figure S3 in Supporting Information. If in systems 6–1–2 and 6–1–5 the spin density on graphene is insignificant, in the other two systems graphene is found to be spin-polarized.

### CO Molecule Adsorbed on Clusters on Graphene

Initially, we have considered the CO molecule adsorbing away from the cluster; however, as it will be clear in the following, the energy of this system is almost 3 eV less favorable than when it is adsorbed on the cluster. Therefore, our next step was to adsorb the molecule within a limited lateral region with the cluster in its center. To this end, we used a modified version of the PSO algorithm to find the lowest energy structures of the CO molecule at or near the 6–1 cluster adsorbed on graphene. The algorithm in this case was modified since the rotation of the adsorbed molecule around its axis is redundant and hence only two angles need to be considered. Here, for simplicity, we fixed the multiplicity to 1 as above and ignored the spin polarization.

**Figure 6:**
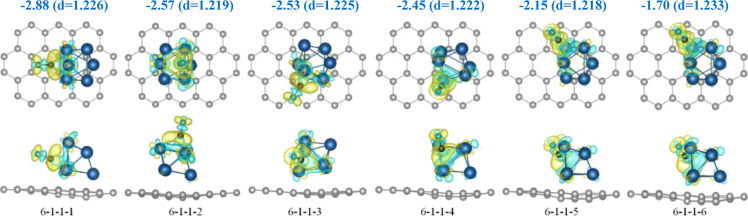
Top (above) and side (below) views of the best six obtained geometries of the CO molecule at or near the 6–1–1 cluster on graphene. The adsorption energies (in eV) and the CO bond lengths *d* (in Å) are indicated. The corresponding charge density difference is also shown. The green and yellow colors correspond to depletion and excess of the density (at ±0.005 Bohr^–3^), respectively.

As in the previous cases, more than 20 adsorption geometries of the CO molecules on the 6–1–1 cluster on graphene were tried in our PSO calculations, with the overall spread of their energies being of 2.67 eV. However, only six geometries within 1.2 eV from the best structure were obtained, they are shown in Figure 6. The binding energies (defined, as before, as the energy difference between the whole system and the two fragments, one being the cluster on graphene and the other the individual CO molecule) and the CO bond length *d* in each case are also shown. Note that the energy of the CO molecule adsorbed away from the cluster was found to be 2.82 eV less favorable than the best structure 6–1–1–1 with the molecule adsorbed on the cluster.

It is seen that the energy difference between the first (the best) and the second configuration is 0.31 eV, which is significant rendering the best structure 6–1–1–1 we found to be the most abundant (statistically relevant). The energy differences between the next four configurations are very small, making them all being almost equally probable. The sixth geometry is by 0.45 eV less favorable than the previous geometry. Considering the energy differences, only the best structure must be of importance in further analysis. However, as an illustration of the possible participation of more than a single initial structure, we have also included the second-best structure 6–1–1–2 in our simulations of the reaction kinetics (see the next section). We shall see that in our particular case, its contribution can safely be neglected unless at very high temperatures (and hence of the other three structures following after it in energy). Of course, more structures will have to be considered, would the energy differences with the best structure be smaller. We also note that in all geometries the CO molecule retains its individuality as its bond length exhibits minimal variation across different configurations.

The charge density differences between the whole system and two its individual parts, the cluster on graphene and the CO molecule, calculated in the geometry of the relaxed complex, for the best six geometries, are also shown in Figure 6. A considerable redistribution of the electron density can be seen due to the bonding of the CO to the cluster in all cases. No redistribution is found between the cluster and graphene. The electronic density is not affected in this region by the adsorption of the CO to the cluster, as one might expect.

### Dissociation of the Molecule

After establishing the two most favorable structures of the CO molecule on the Mo_6_ cluster adsorbed on graphene, we can consider the CO dissociation reaction. We start by establishing a set of possible final geometries after the CO dissociation. To this end, we ran a simple version of the PSO algorithm that was concerned in each case only with the lateral positions of the O atom of the molecule near or at the cluster, i.e., we consider only two degrees of freedom. The *z* coordinate of the O atom was in all cases initially taken at random between 1.6 and 2.1 Å above the highest atom of the cluster, prior to geometry optimization. For simplicity, the same initial position of the C atom was used in these calculations. (Of course, in an exhaustive analysis we would have to consider all possible separate positions of the C and O atoms as the final states of the dissociation reaction; this will be left for future studies.) In addition, we have also considered the molecule to dissociate on graphene away from the cluster. However, the energy of the final structure was found to be even less favorable (by 6.76 eV) than the energy of the CO molecule adsorbed on graphene. Hence, this mechanism for the CO dissociation can be safely disregarded.

We shall start by considering possible reaction paths from the best 6–1–1–1 geometry. Overall, as the final geometry, 12 positions of the O atom were found with the spread of energies being 0.55 eV. Only the best two such structures will be further explored here.

In the first structure (numbered 4), we found the O atom being on top of the cluster with the energy by 0.67 eV lower than in the initial state (numbered 1), and in the second structure (numbered 6) the O atom is found at the other side of the cluster with the energy being lowered by further 0.33 eV. This latter structure has the lowest energy we find in it C and O atoms are positioned at the opposite sides of the cluster.

**Figure 7:**
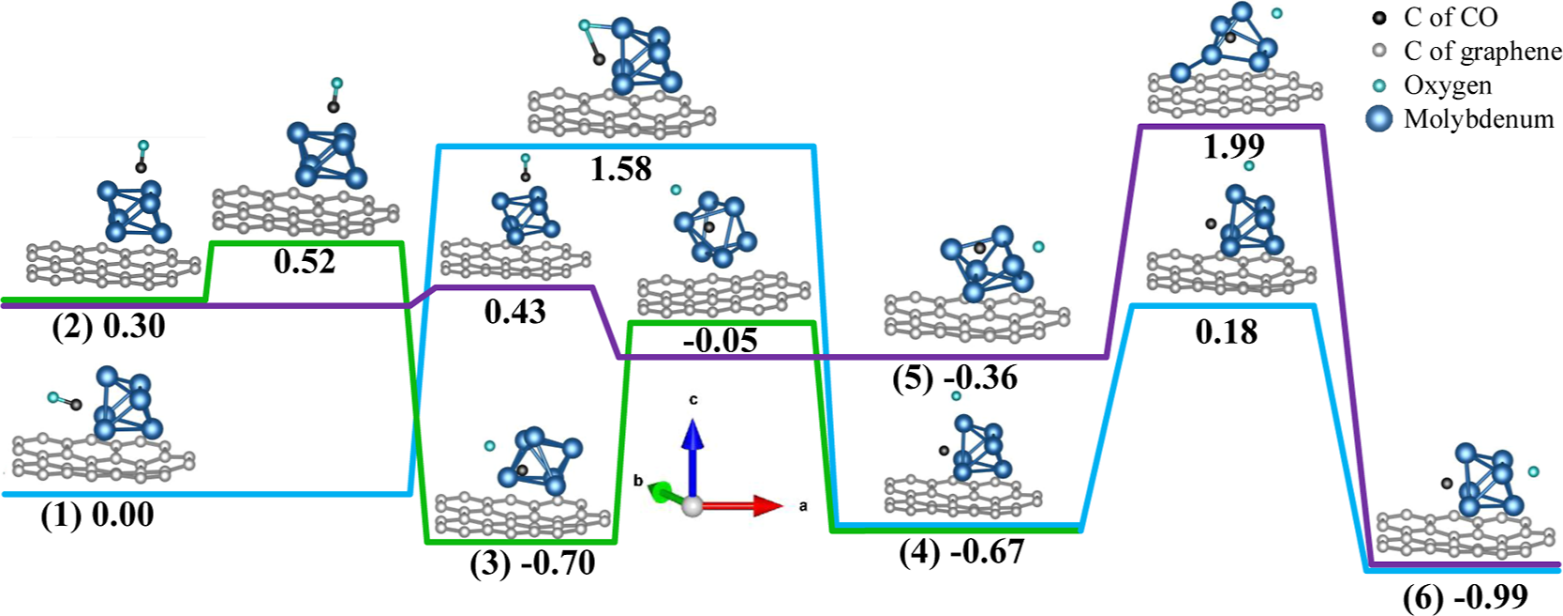
CO molecule dissociation kinetics modeled from two different initial structures. Relative energies (in eV) of each configuration with respect to the structure 1 are also shown. Note that different states of the system shown by horizontal lines are not drawn to the correct energy scale. All six geometries are numbered as in eq 3.

Initially, two NEB simulations were run, one between states 1 and 4 (3 images between the initial and final states), and another—between states 4 and 6 (4 images), see the blue curve in Figure 7. The dissociation is governed by the first transition (1 → 4) with the barrier of 1.58 eV as the second barrier for the 4 → 6 transition is much lower (0.85 eV).

Next, we performed dissociation calculations from the second-best initial state 6–1–1–2 of the CO molecule on the cluster (numbered 2). By running an NEB simulation between states 2 and 6 we found, however, a new intermediate state (numbered 5), and therefore had to split the calculation into two, 2 → 5 (the barrier 0.13 eV, the number of images used is 6) and 5 → 6 (4 images with the barrier of 2.35 eV), see the purple curve in Figure 7.

Finally, attempts have been made to run NEB simulations between the two initial states and various intermediate minima we have found. Only one such calculation, namely between states 2 and 4, was successful, the green curve in Figure 7, after it was split into two separate simulations (using by 6 images in either case) employing an additional intermediate state 3 that is lower than the best initial state by 0.7 eV. Other calculations resulted in very large barriers (over 3 eV) due to considerable reorganization of the cluster and both C and O atoms that was required; we do not show these simulations here. Interestingly, geometry 3 did not come out in our PSO simulations; this must be due to an insufficient number of generations used.

Therefore, considering two initial geometries of the CO molecule on the Mo_6_ cluster, we obtained four stable final geometries of the dissociated molecule. To determine the expected populations of the latter geometries after the reaction (at long times), a simple rate equation analysis can be performed. Let *N*_1_(*t*) to *N*_6_(*t*) be populations of the two initial (the CO molecule is not yet dissociated) and the four final structures (C and O atoms are separated), respectively. *N*_6_ corresponds to the lowest energy dissociated state of the CO molecule. These populations can be obtained by solving the system of linear rate equations
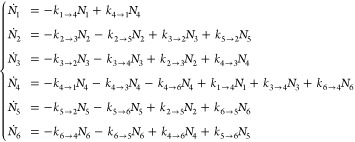
3where  is the reaction rate from structure *i* to *j* (=1,···, 6), Δ*E*_*i*→*j*_ the corresponding energy barrier deduced from Figure 7 and β = 1/*k*_B_*T*, where *k*_B_ is the Boltzmann’s constant. All the barriers are shown in Table 2. It follows from the structure of the model that, as expected, at long times the concentrations will approach their solutions obtained by setting the left-hand sides of the equations to zero and employing the conservation condition ∑_*i*=1_^6^*N*_*i*_(*t*) = 1 valid at any time *t*.

**Table 2:** Calculated Energy Barriers for the Specified Transitions

color in Figure 7	transition	barrier (eV)
blue	1 → 4	1.58
	4 → 1	2.25
	4 → 6	0.85
	6 → 4	1.17
green	2 → 3	0.22
	3 → 2	1.22
	3 → 4	0.65
	4 → 3	0.62
purple	2 → 5	0.13
	5 → 2	0.79
	5 → 6	2.35
	6 → 5	2.98

In practical calculations, we have used the same prefactor ν = 10^13^ s^–1^, the characteristic vibrational frequency. The prefactors can be calculated, within the classical transition state theory, by calculating vibrational frequencies in the minima and the saddle points;^61^ however, these calculations are nontrivial for complex systems as require the precise location of these geometries. Hence these were not attempted here as the exact values of the prefactors usually have only a minor influence on the results.

**Figure 8:**
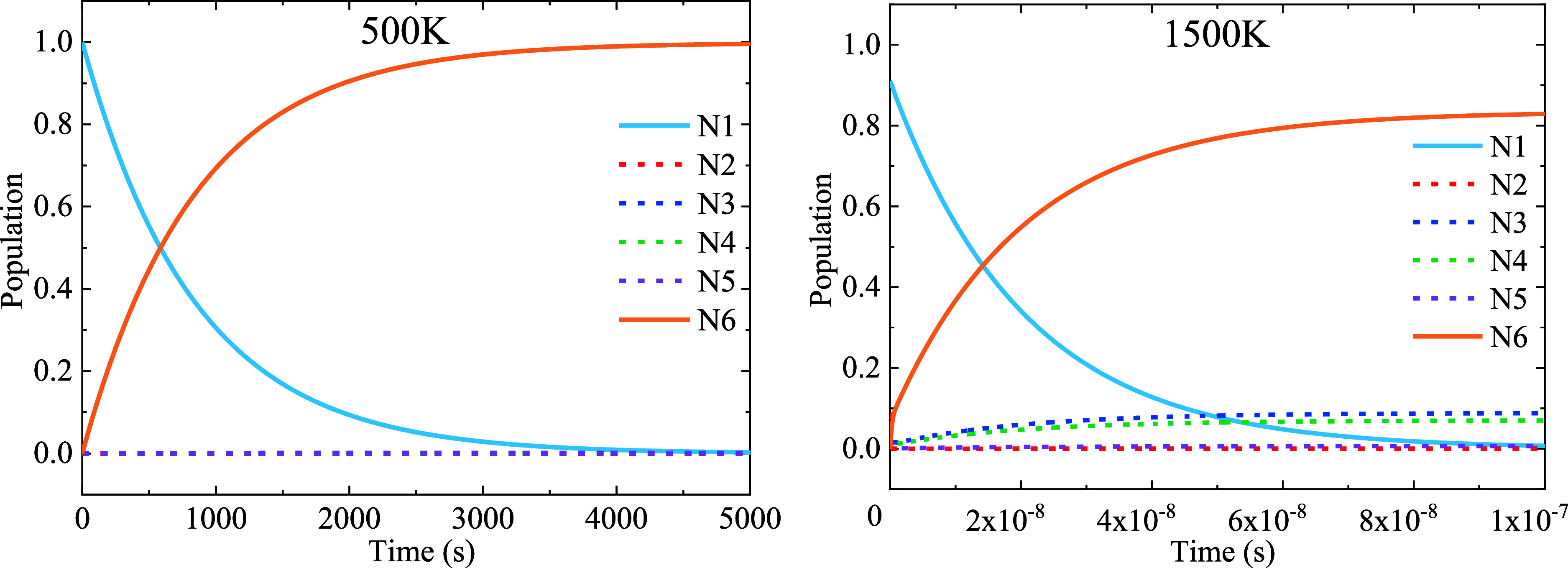
Time dependence (in sec) of the populations of the CO, *N*_1_(*t*) and *N*_2_(*t*), and of the products, *N*_*i*_(*t*) (*i* = 3,···, 6), during the course of the CO dissociation reaction.

Initially, the populations of the intermediate and final states are set to zero. The initial populations of the two initial states *N*_1_(0) and *N*_2_(0) are chosen using the canonical distribution
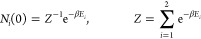
4where *E*_*i*_ is the binding energy of the *i*-th geometry (*i* = 1, 2). Clearly, only relative energies are required. We neglected here possible kinetic transformations of the nanocluster (the so-called fluxionality effect^18,37,59^) assuming its lifetime is longer than the characteristic reaction times (see Figure 8).

Solving the set of eq 3 subject to the mentioned initial conditions, we obtain the time dependence of the populations as shown in Figure 8 for two temperatures, 500 and 1500 K.

At 500 K, we observe that practically all CO molecules dissociate, with the C and O atoms being at the opposite sides of the cluster, state 6. This process takes over an hour. The populations of the intermediate states, as expected, are found negligible. At 1500 K only just over 80% of the molecules dissociate into state 6, with some small, but noticeable, populations found of the intermediate states 3 and 4. The whole process at this temperature takes less than a microsecond. Similar results are obtained for 1000 K, albeit with the reaction taking about 600 ms instead (not shown).

The obtained results are to be expected due to a relatively large energy barrier (of over 1.5 eV) found in our NEB simulations for breaking the CO bond on the cluster (departure from state 1): the dissociation of CO molecules can only be observed at high enough temperatures. Still, the catalytic effect of the Mo clusters becomes evident if this barrier is compared with the CO dissociation energy of being over 11 eV.^72^ Also, as expected, the role of the second best initial state starts playing some (still rather marginal) role only at high enough temperatures: if at 500 K the relative initial population of state 2 is only 0.1%, at 500 K it grows to 3% and at 1500 K to 9%. These results are broadly in agreement with the observations^73^ that CO molecules are not broken down by Molybdenum nanoclusters adsorbed on a thin alumina film unless one goes to annealing temperatures of the order of 700 K. Also, in DFT calculations^44^ rather large dissociation barrier was obtained for the CO dissociation on the corrugated Mo(112) surface. Interestingly, CO molecules were observed to dissociate, e.g., on the flat Mo(110) surface even at temperatures as low as 125 K.^49^ Hence, the ability of the Mo systems to split the CO molecule is dependent on the actual Mo system, specifically, on the coordination of the Mo atoms.

## Conclusion and Discussion

In this work, we proposed a systematic global optimization search-based computational strategy for studying catalytic properties of subnanometer clusters (SNCs) on a crystal surface in nanocluster beam experiments. The strategy consists of several well-defined stages: (i) determine the most stable clusters on the surface of interest; this step would most likely be influenced by the actual experimental procedure; note that more than one close-in-energy structure may come out of this analysis, all need to be accounted for; (ii) for each of the possibilities found in (i) consider all possibilities for the chemical reaction of interest catalyzed by the clusters; (iii) simulate the populations of the reactants and products after the reactions (at long times) taking into account the multitude of reaction paths from (ii).

Even though this strategy must have already been followed in the literature and is well-known, the message that we have tried to communicate here is that at each stage of this procedure, there are multitudes of possibilities that are rarely (if at all) considered in practical simulations, so that one has to use, at each stage, appropriate stochastic methods to achieve a proper sampling to account for all these possibilities. This point is highlighted by the large number of stable structures we have found with varying energies emphasizing the need for careful initial geometry selection. A “common sense” approach might lead to structures that are less relevant (e.g., of higher free energy). Note that we have not attempted to carry out free energy calculations here due to the high computational cost; only DFT total energies were computed.

To illustrate our strategy, we considered in detail the catalytic properties of Mo_6_ clusters adsorbed on the free-standing graphene with respect to the CO molecule dissociation reaction. Specifically, as an experimental procedure, we have examined the beam deposition method^74^ in which clusters of a precise number of atoms are evaporated on a surface from a gun without their destruction upon impact. Note that the particular system we have chosen, Molybdenum clusters on the free-standing graphene, may not be of significant practical interest (even though Molybdenum is well-known for its catalytic properties^40−42^); it was used here on computational grounds only to illustrate the strategy. However, the systems of real interest might be a straightforward extension; for instance, instead of the free-standing graphene, one may consider adsorption of Mo clusters on a graphene attached to a transition metal surface;^12,75,76^ these systems are however computationally much more demanding and are left for future work.

Note that in performing our simulations, we have assumed a uniform distribution of the clusters in the beam. This has been done solely to simplify the computational approach. If one wishes to get beyond this approximation, it is necessary to take account of the interactions between the clusters in the beam and compute their actual trajectories.^77^

Our adapted strategy for this system involved: (i) identifying the lowest-energy (positively charged) Mo clusters using AIRSS, (ii) determining their optimal adsorption geometries on graphene using PSO, (iii) finding the lowest-energy CO adsorption sites on the most energetically favorable Mo cluster also using PSO, (iv) identifying possible final states (products) after CO dissociation near the surface employing a simplified version of PSO, (v) calculating energy barriers using NEB simulations to determine the transition rates between the initial and final states for all minimum energy paths connecting all selected initial and final states and (vi) performing rate equations analysis to determine the time evolution of populations of different states. For CO on a Mo_6_ cluster on graphene, we found high temperatures are needed for dissociation, which broadly agrees with experiment^73^ (although conducted for a different support). Finally, we showed that CO dissociation is unlikely to occur directly on graphene, highlighting the crucial role of Mo clusters in catalyzing this reaction.

The main message we want to deliver is that to ensure reliable computational results in catalysis, stochastic methods are necessary at each stage to properly sample the configurational space and identify all relevant low-energy structures. A simple initial geometry guess can lead to unrepresentative structures and misleading conclusions about catalytic properties.

Another point worth mentioning is that in some cases metastable configurations may be even more important when considering a given reaction, than the global minimum one.Indeed, transition rates out of some metastable states of the reactants could be higher than out of the global minimum state (the barriers lower), so that the former, not the latter, would mostly contribute to the reaction whereby exhibiting unique catalytic properties.^37^ Therefore, when applying global search stochastic methods, it is essential to keep also higher energy structures and consider their role in catalysis. Two points are in order here: (i) initial populations of metastable states crucially depend on their relative energies (with respect to the global minimum state), and (ii) one may need to take account of the dynamics of reactants as well in case their lifetimes are shorter than the characteristic time of the reaction itself.^59^ We have not considered the second point in this work assuming that our Mo clusters are sufficiently stable; however, as an example, we inspected the first point by considering the second most favorable CO configuration on the cluster and its role in the dissociation dynamics. Even though in our case the dynamics is driven by the most favorable CO configuration, this does not need to be the case in general.

It is appreciated that any method related to global search optimization is computationally expensive as it is based on screening very many structures. If a DFT based method is employed to perform total energy relaxation calculations and a modest computational resource is available, then inevitably one would only be limited to relatively small systems. If bigger systems are of interest, then approaches that make the calculations more efficient must be sought. In particular, methods based on Machine Learning^78,79^ or relying on force fields of ab initio quality^80^ must be considered as the most important practical ingredient of the proposed approach. Importantly, however, the general computational strategy outlined in this work would remain the same.

We hope that this study will be of utility to a wide class of researchers in computational surface physics and chemistry.

## Supporting Information Available

Generating cluster geometry on graphene in PSO method; Adsorption results based on the best neutral Mo_6_ cluster; Spin density results based on the best charged Mo_6_ cluster; The full path for NEB calculation (PDF)

## References

[ref1] James T. E.; Hemmingson S. L.; Campbell C. Energy of Supported Metal Catalysts: From Single Atoms to Large Metal Nanoparticles. ACS Catal. 2015, 5, 5673–5678. 10.1021/acscatal.5b01372.

[ref2] Campelo J. M.; Luna D.; Luque R.; Marinas J. M.; Romero A. A. Sustainable preparation of supported metal nanoparticles and their applications in catalysis. ChemSusChem 2009, 2, 18–45. 10.1002/cssc.200800227. 19142903

[ref3] Lu Y.; Chen W. Sub-nanometre sized metal clusters: from synthetic challenges to the unique property discoveries. Chem. Soc. Rev. 2012, 41, 3594–3623. 10.1039/c2cs15325d. 22441327

[ref4] Chen W.; Chen S. Oxygen Electroreduction Catalyzed by Gold Nanoclusters: Strong Core Size Effects. Angew. Chem., Int. Ed. 2009, 48, 4386–4389. 10.1002/anie.200901185. 19431173

[ref5] Muhammed M. A. H.; Verma P. K.; Pal S. K.; Kumar R. A.; Paul S.; Omkumar R. V.; Pradeep T. Bright, NIR-emitting Au23 from Au25: characterization and applications including biolabeling. Chem.—Eur. J. 2009, 15, 10110–10120. 10.1002/chem.200901425. 19711391

[ref6] Santiago Gonzalez B.; Rodriguez M. J.; Blanco C.; Rivas J.; López-Quintela M. A.; Martinho J. M. G. One step synthesis of the smallest photoluminescent and paramagnetic PVP-protected gold atomic clusters. Nano Lett. 2010, 10, 4217 10.1021/nl1026716. 20836542

[ref7] Fernández E.; Boronat M. Sub nanometer clusters in catalysis. J. Phys.: Condens.Matter 2019, 31, 013002 10.1088/1361-648X/aaed84. 30499451

[ref8] Zhang B.; Chen Y.; Wang J.; Pan H.; Sun W. Supported Sub-Nanometer Clusters for Electrocatalysis Applications. Adv. Funct. Mater. 2022, 32, 2202227 10.1002/adfm.202202227.

[ref9] Pacchioni G. Electronic interactions and charge transfers of metal atoms and clusters on oxide surfaces. Phys. Chem. Chem. Phys. 2013, 15, 1737–1757. 10.1039/c2cp43731g. 23287900

[ref10] Wegner K.; Piseri P.; Tafreshi H. V.; Milani P. Cluster beam deposition: a tool for nanoscale science and technology. J. Phys. D: Appl. Phys. 2006, 39, R439–R459. 10.1088/0022-3727/39/22/R02.

[ref11] Halder A.; Curtiss L. A.; Fortunelli A.; Vajda S. Perspective: Size selected clusters for catalysis and electrochemistry. J. Chem. Phys. 2018, 148, 110901 10.1063/1.5020301. 29566496

[ref12] Loi F.; Pozzo M.; Sbuelz L.; Bignardi L.; Lacovig P.; Tosi E.; Lizzit S.; Kartouzian A.; Heiz U.; Alfè D.; Baraldi A. Oxidation at the sub-nanoscale: oxygen adsorption on graphene-supported size-selected Ag clusters. J. Mater. Chem. 2022, 10, 14594–14603. 10.1039/d2ta02539f.

[ref13] Huang X.; Lu R.; Cen Y.; Wang D.; Jin S.; Chen W.; Geoffrey I.; Waterhouse N.; Wang Z. S. T.; Tian S.; et al. Micropore-confined Ru nanoclusters catalyst for efficient pH-universal hydrogen evolution reaction. Nano Res. 2023, 16, 9073–9080. 10.1007/s12274-023-5711-1.

[ref14] Ghosh A.; Sagadevan A.; Murugesan K.; Nastase S. A. F.; Maity B.; Bodiuzzaman M.; Shkurenko A.; Hedhili M. N.; Yin J.; Mohammed O. F.; Eddaoudi M.; Cavallo L.; Rueping M.; Bakr O. M. Multiple neighboring active sites of an atomically precise copper nanocluster catalyst for efficient bond-forming reactions. Mater. Horiz. 2024, 11, 2494–2505. 10.1039/D4MH00098F. 38477151

[ref15] Zaman N.; Lasri K.; Lau K. C.; Amine K.; Kara A. Computational study of the adsorption of bimetallic clusters on alumina substrate. Surf. Sci. 2020, 700, 121682 10.1016/j.susc.2020.121682.

[ref16] Jin C.; Cheng L.; Feng G.; Ye R.; Lu Z.-H.; Zhang R.; Yu X. Adsorption of Transition-Metal Clusters on Graphene and N-Doped Graphene: A DFT Study. Langmuir 2022, 38, 3694–3710. 10.1021/acs.langmuir.1c03187. 35285652

[ref17] Hao X.; Zhang R.; He L.; Huang Z.; Wang B. Coverage-dependent adsorption, dissociation and aggregation of H2O on the clean and pre-adsorbed oxygen Cu (111) surface: A DFT study. Mol. Catal. 2018, 445, 152–162. 10.1016/j.mcat.2017.11.034.

[ref18] Ghosh P.; Camellone M. F.; Fabris S. Fluxionality of Au clusters at ceria surfaces during CO oxidation: Relationships among reactivity, size, cohesion, and surface defects from DFT simulations. J. Chem. Phys. Lett. 2013, 4, 2256–2263. 10.1021/jz4009079.

[ref19] Zhang M.; Mao Y.; Bao X.; Wang P.; Liu Y.; Zheng Z.; Cheng H.; Dai Y.; Wang Z.; Huang B. Promoting Photocatalytic CO2Methanation by the Construction of Cooperative Copper Dual-Active Sites. ACS Catal. 2024, 14, 5275–5285. 10.1021/acscatal.4c00060.

[ref20] Chen S.; Zheng F.; Wu S.; Zhu Z. An improved genetic algorithm for crystal structure prediction. Curr. Appl. Phys. 2017, 17, 454–460. 10.1016/j.cap.2017.01.010.

[ref21] Deaven D. M.; Ho K.-M. Molecular geometry optimization with a genetic algorithm. Phys. Rev. Lett. 1995, 75, 288–291. 10.1103/PhysRevLett.75.288. 10059656

[ref22] Davis J. B. A.; Shayeghi A.; Horswell S. L.; Johnston R. L. The Birmingham parallel genetic algorithm and its application to the direct DFT global optimization of Ir_N_ (N = 10–20) clusters. Nanoscale 2015, 7, 14032 10.1039/C5NR03774C. 26239404

[ref23] Kanters R. P. F.; Donald K. J. CLUSTER: Searching for unique low energy minima of structures using a novel implementation of a genetic algorithm. J. Chem. Theory Comput. 2014, 10, 5729–5737. 10.1021/ct500744k. 26583254

[ref24] Alexandrova A. N. H·(H_2_O)_n_ clusters: Microsolvation of the hydrogen atom via molecular ab initio gradient embedded genetic algorithm (GEGA). J. Phys. Chem. A 2010, 114, 12591 10.1021/jp1092543. 21077611

[ref25] Liu S.; Zong J.; Zhao Z. J.; Gong J. Exploring the initial oxidation of Pt, Pt3Ni, Pt3Au (111) surfaces: a genetic algorithm based global optimization with density functional theory. Greeen. Chem. Eng. 2020, 1, 56 10.1016/j.gce.2020.09.006.

[ref26] Heard C. J.; Heiles S.; Vajda S.; Johnston R. L. Pd_n_Ag_(4–n)_ and Pd_n_Pt_(4–n)_ clusters on MgO (100): a density functional surface genetic algorithm investigation. Nanoscale 2014, 6, 11777–11788. 10.1039/c4nr03363a. 25158024

[ref27] Pickard C. J.; Needs R. High-pressure phases of silane. Phys. Rev. Lett. 2006, 97, 045504 10.1103/PhysRevLett.97.045504. 16907590

[ref28] Pickard C. J.; Needs R. J. Structures at high pressure from random searching. Phys. Status Solidi B 2009, 246, 536–540. 10.1002/pssb.200880546.

[ref29] Pickard C. J.; Needs R. Ab initio random structure searching. J. Phys.: Condens.Matter 2011, 23, 053201 10.1088/0953-8984/23/5/053201. 21406903

[ref30] Fischer C. C.; Tibbetts K. J.; Morgan D.; Ceder G. Predicting crystal structure by merging data mining with quantum mechanics. Nat. Mater. 2006, 5, 641–646. 10.1038/nmat1691. 16845417

[ref31] Pannetier J.; Bassas-Alsina J.; Rodriguez-Carvajal J.; Caignaert V. Prediction of crystal structures from crystal chemistry rules by simulated annealing. Nature 1990, 346, 343–345. 10.1038/346343a0.

[ref32] Wang J.; Ma L.; Zhao J.; Jackson K. A. Structural growth behavior and polarizability of CdnTen (n= 1–14) clusters. J. Chem. Phys. 2009, 130, 214307 10.1063/1.3147519. 19508069

[ref33] Wales D. Energy Landscapes: Applications to Clusters, Biomolecules and Glasses; Cambridge Univ. Press, 2004.

[ref34] Wang Y.; Lv J.; Zhu L.; Ma Y. CALYPSO: A method for crystal structure prediction. Comput. Phys. Commun. 2012, 183, 2063–2070. 10.1016/j.cpc.2012.05.008.

[ref35] Modern methods of crystal structure prediction; Oganov A. R., Ed.; Wiley, 2010.

[ref36] Zhai H.; Hao H.; Yeo J. Benchmarking inverse optimization algorithms for materials design. APL Mater. 2024, 12, 021107 10.1063/5.0177266.

[ref37] Zandkarimi B.; Alexandrova A. N. Surface-supported cluster catalysis: Ensembles of metastable states run the show. Wiley Interdiscipl. Reviews: Comput. Mol. Sci. 2019, 9, e1420 10.1002/wcms.1420.

[ref38] Wang D.; Tan D.; Liu L. Particle swarm optimization algorithm: an overview. Soft Comput. 2018, 22, 387–408. 10.1007/s00500-016-2474-6.

[ref39] Shi Y. Particle swarm optimization. IEEE connections 2004, 2, 8–13.

[ref40] Hua W.; Sun H.-H.; Xu F.; Wang J.-G. A review and perspective on molybdenum-based electrocatalysts for hydrogen evolution reaction. Rare Met 2020, 39, 335–351. 10.1007/s12598-020-01384-7.

[ref41] Zhou Z.; Jia Y.; Wang Q.; Jiang Z.; Xiao J.; Guo L. Recent Progress on Molybdenum Carbide-Based Catalysts for Hydrogen Evolution: A Review. Sustainability 2023, 15, 14556 10.3390/su151914556.

[ref42] Ji J.; Bao Y.; Liu X.; Zhang J.; Xing M. Molybdenum-based Heterogeneous Catalysis for the Control of Environmental Pollutants; EcoMat, 2021; p e12155.

[ref43] Juel M.; Raaen S. Thermal desorption of carbon monoxide from Mo(110). Philos. Mag. 2003, 83, 2475–2486. 10.1080/1478643031000116023.

[ref44] Yakovkin I. N.; Petrova N. V. Absence of CO dissociation on Mo(112). J. Chem. Phys. 2009, 130, 174714 10.1063/1.3126774. 19425805

[ref45] Yang T.-s.; Jee H.-g.; Boo J.-H.; Kim Y. D.; Lee S.-B. CO Adsorption on Mo(110) Studied Using Thermal Desorption Spectroscopy (TDS) and Ultraviolet Photoelectron Spectroscopy (UPS). Bull. Korean Chem. Soc. 2009, 30, 1353.

[ref46] Raaen S.; Yu X. Temperature programmed desorption of CO from CO pre-covered Mo(110). Appl. Surf. Sci. 2015, 349, 17–20. 10.1016/j.apsusc.2015.04.186.

[ref47] Tian X.; Wang T.; Jiao H. Mechanism of coverage dependent CO adsorption and dissociation on the Mo(100) surface. Phys. Chem. Chem. Phys. 2017, 19, 2186–2192. 10.1039/C6CP08129K. 28045154

[ref48] Gleichweit C.; Neiss C.; Maisel S.; Bauer U.; Späth F.; Höfert O.; Görling A.; Steinrück H. P.; Papp C. Surface Reaction of CO on Carbide-Modified Mo(110). J. Phys. Chem. C 2017, 121, 3133–3142. 10.1021/acs.jpcc.6b11950.

[ref49] Jaworowski A.; Smedh M.; Borg M.; Sandell A.; Beutler A.; Sorensen S.; Lundgren E.; Andersen J. CO dissociation on Mo(110) studied by high-resolution core-level spectroscopy. Surf. Sci. 2001, 492, 185–194. 10.1016/S0039-6028(01)01447-9.

[ref50] Cox D. M.; Reichmann K. C.; Trevor D. J.; Kaldor A. CO chemisorption on free gas phase metal clusters. J. Chem. Phys. 1988, 88, 111–119. 10.1063/1.454643.

[ref51] Addicoat M. A.; Buntine M. A.; Yates B.; Metha G. F. Associative versus dissociative binding of CO to 4d transition metal trimers: A density functional study. J. Comput. Chem. 2008, 29, 1497–1506. 10.1002/jcc.20912. 18393258

[ref52] Heinrich R.; Wucher A. Cluster formation under bombardment with polyatomic projectiles. Nucl. Instrum. Methods Phys. Res., Sect. B 2000, 164–165, 720–726. 10.1016/S0168-583X(99)01118-0.

[ref53] Li L.; Li H.; Zeng X. C. Structure transition of Au 18 from pyramidal to a hollow-cage during soft-landing onto a TiO 2 (110) surface. Chem. Commun. 2015, 51, 9535–9538. 10.1039/C5CC01316J. 25969847

[ref54] Popok V. N.; Barke I.; Campbell E. E.; Meiwes-Broer K.-H. Cluster–surface interaction: From soft landing to implantation. Surf. Sci. Rep. 2011, 66, 347–377. 10.1016/j.surfrep.2011.05.002.

[ref55] Johnson G. E.; Gunaratne D.; Laskin J. Soft-and reactive landing of ions onto surfaces: Concepts and applications. Mass Spectrom. Rev. 2016, 35, 439–479. 10.1002/mas.21451. 25880894

[ref56] Seki K.; Wojcik M.; Tachiya M. Fractional reaction-diffusion equation. J. Chem. Phys. 2003, 119, 2165–2170. 10.1063/1.1587126.

[ref57] Hellander S.; Hellander A.; Petzold L. Reaction rates for mesoscopic reaction-diffusion kinetics. Phys. Rev. E 2015, 91, 023312 10.1103/PhysRevE.91.023312. PMC4854576 25768640

[ref58] Tetlow H.; Curcio D.; Baraldi A.; Kantorovich L. Hydrocarbon decomposition kinetics on the Ir(111) surface. Phys. Chem. Chem. Phys. 2018, 20, 6083–6099. 10.1039/c7cp07526j. 29303172

[ref59] Poths P.; Vargas S.; Sautet P.; Alexandrova A. N. Thermodynamic Equilibrium versus Kinetic Trapping: Thermalization of Cluster Catalyst Ensembles Can Extend Beyond Reaction Time Scales. ACS Catal. 2024, 14, 5403–5415. 10.1021/acscatal.3c06154.

[ref60] Fichthorn K. A.; Weinberg W. H. Theoretical foundations of dynamical Monte Carlo simulations. J. Chem. Phys. 1991, 95, 1090–1096. 10.1063/1.461138.

[ref61] Nitzan A. Chemical Dynamics in Condensed Phases: Relaxation, Transfer, and Reactions in Condensed Molecular Systems; Oxford University Press, 2014.

[ref62] Tetlow H.; Posthuma de Boer J.; Ford I. J.; Vvedensky D. D.; Curcio D.; Omiciuolo L.; Lizzit S.; Baraldi A.; Kantorovich L. Ethylene decomposition on Ir(111): initial path to graphene formation. Phys. Chem. Chem. Phys. 2016, 18, 27897–27909. 10.1039/c6cp03638d. 27711652

[ref63] Kresse G.; Furthmüller J. Efficient iterative schemes for ab initio total-energy calculations using a plane-wave basis set. Phys. Rev. B 1996, 54, 11169–11186. 10.1103/PhysRevB.54.11169. 9984901

[ref64] Kresse G.; Furthmüller J. Efficiency of ab-initio total energy calculations for metals and semiconductors using a plane-wave basis set. Comput. Mater. Sci. 1996, 6, 15–50. 10.1016/0927-0256(96)00008-0. 9984901

[ref65] Perdew J. P.; Burke K.; Ernzerhof M. Generalized gradient approximation made simple. Phys. Rev. Lett. 1996, 77, 3865–3868. 10.1103/PhysRevLett.77.3865. 10062328

[ref66] Grimme S.; Ehrlich S.; Goerigk L. Effect of the damping function in dispersion corrected density functional theory. J. Comput. Chem. 2011, 32, 1456–1465. 10.1002/jcc.21759. 21370243

[ref67] Civalleri B.; Zicovich-Wilson C. M.; Valenzano L.; Ugliengo P. B3LYP augmented with an empirical dispersion term (B3LYP-D*) as applied to molecular crystals. CrystEngComm 2008, 10, 405–410. 10.1039/B715018K.

[ref68] Wei Y.; Veryazov V.; Kantorovich L. A comprehensive exploration of structural and electronic properties of molybdenum clusters. APL Mater. 2024, 12, 031127 10.1063/5.0197987.

[ref69] Henkelman G.; Jónsson H. Improved tangent estimate in the nudged elastic band method for finding minimum energy paths and saddle points. J. Chem. Phys. 2000, 113, 9978–9985. 10.1063/1.1323224.

[ref70] Henkelman G.; Uberuaga B. P.; Jónsson H. A climbing image nudged elastic band method for finding saddle points and minimum energy paths. J. Chem. Phys. 2000, 113, 9901–9904. 10.1063/1.1329672.

[ref71] Zhai H.; Alexandrova A. N. Ensemble-average representation of Pt clusters in conditions of catalysis accessed through GPU accelerated deep neural network fitting global optimization. J. Chem. Theory Comput. 2016, 12, 6213 10.1021/acs.jctc.6b00994. 27951667

[ref72] Gong S.; Wang P.; Mo Y. Measuring the strongest chemical bond with spectroscopic accuracy: CO bond-dissociation energy via predissociation of superexcited states. Phys. Rev. A 2023, 108, 042802 10.1103/PhysRevA.108.042802.

[ref73] Jiang Z.; Huang W.; Zhang Z.; Zhao H.; Tan D.; Bao X. Multiple Coordination of CO on Molybdenum Nanoparticles: Evidence for Intermediate Mo*x*(CO)*y*Species by XPS and UPS. J. Phys. Chem. B 2006, 110, 26105–26113. 10.1021/jp065293i. 17181264

[ref74] Pozzo M.; Turrini T.; Bignardi L.; Lacovig P.; Lizzit D.; Tosi E.; Lizzit S.; Baraldi A.; Alfe D.; Larciprete R. Interplay among Hydrogen Chemisorption, Intercalation, and Bulk Diffusion at the Graphene-Covered Ni (111) Crystal. J. Phys. Chem. C 2023, 127, 6938–6947. 10.1021/acs.jpcc.3c00291.

[ref75] Loi F.; Pozzo M.; Sbuelz L.; Bignardi L.; Lacovig P.; Tosi E.; Lizzit S.; Kartouzian A.; Heiz U.; Larciprete R.; Alf è D.; Baraldi A. Breakdown of the correlation between oxidation states and core electron binding energies at the sub-nanoscale. Appl. Surf. Sci. 2023, 619, 156755 10.1016/j.apsusc.2023.156755.

[ref76] Perco D.; Loi F.; Bignardi L.; Sbuelz L.; Lacovig P.; Tosi E.; Lizzit S.; Kartouzian A.; Heiz U.; Baraldi A. The highest oxidation state observed in graphene- supported sub-nanometer iron oxide clusters. Comm. Chem. 2023, 6, 61 10.1038/s42004-023-00865-x. PMC10070315 37012362

[ref77] De Vita A.; Štich I.; Gillan M. J.; Payne M. C.; Clarke L. J. Dynamics of dissociative chemisorption: Cl_2_/Si(111)-(2 × 1). Phys. Rev. Lett. 1993, 71, 1276 10.1103/PhysRevLett.71.1276. 10055495

[ref78] Ma S.; Liu Z.-P. Machine learning for atomic simulation and activity prediction in heterogeneous catalysis: current status and future. ACS Catal. 2020, 10, 13213–13226. 10.1021/acscatal.0c03472.

[ref79] Toyao T.; Maeno Z.; Takakusagi S.; Kamachi T.; Takigawa I.; Shimizu K.-i. Machine learning for catalysis informatics: recent applications and prospects. ACS Catal. 2020, 10, 2260–2297. 10.1021/acscatal.9b04186.

[ref80] Deringer V. L.; Caro M. A.; Csányi G. A general-purpose machine-learning force field for bulk and nanostructured phosphorus. Nat. Comm. 2020, 11, 5461 10.1038/s41467-020-19168-z. PMC7596484 33122630

