# Supplementary material for: Global Optimization of Molybdenum Subnanoclusters on Graphene: A Consistent Approach toward Catalytic Applications
Source: ACS Appl Mater Interfaces. 2024 Nov 6;16(46):64177–89. doi: 10.1021/acsami.4c13102 (PMC11583127; doi:10.1021/acsami.4c13102)
Supplement: Supplementary file 1 — am4c13102_si_001.pdf [file am4c13102_si_001.pdf]

Supporting Information

Global Optimization of Molybdenum Subnanoclusters on Graphene: a Consistent Approach Towards Catalytic Applications

Yao Wei,[1] Alejandro Santana-Bonilla,[1] and Lev Kantorovich[1,*]

[1] *Theory and Simulation of Condensed Matter (TSCM), King's College London, Strand, London WC2R 2LS, United Kingdom*

* lev.kantorovitch@kcl.ac.uk

I. GENERATING CLUSTER GEOMETRY ON GRAPHENE IN PSO METHOD

This work establishes a direct method for deriving the atomic positions of Mo clusters on graphene utilizing the five-dimensional search space inherent in the Particle Swarm Optimization (PSO) algorithm. The degrees of freedom can be split into 2 lateral positions of the cluster's centre of mass (X_{cm} and Y_{cm}) and 3 Euler's angles (u, v and w) represent the possible rotations in the space (see Table SI). The vertical (Z_{cm}) position of the cluster's centre of mass is defined such that the distance between the unrelaxed graphene sheet and the lowest atom of the cluster (for the given orientation) is 3.0 Å. This is sufficient for the cluster to experience a noticeable vertical force from graphene.

TABLE SI: Euler angles.

**Axis of rotation**	**Euler angle name**	**Euler angle symbol**
x	Roll	u
y	Pitch	v
z	Yaw	w

If $\mathbf{R}_0 = (x_0, y_0, z_0)$ are Cartesian coordinates of an atom of the cluster in the laboratory coordinate system in which the cluster is defined in the gas phase, with its centre of mass being at the zero point, then the coordinates $\mathbf{R} = (x, y, z)$ of that atom after rotation by the three Euler angles and translation by the vector $\mathbf{R}_{cm} = (X_{cm}, Y_{cm}, Z_{cm})$ are found as

$$\mathbf{R} = \mathbf{R}_{cm} + \mathbf{U}_z(w)\mathbf{U}_y(v)\mathbf{U}_x(u)\mathbf{R}_0 \,,$$

where the three rotations around the x, y and z axes are defined by their respective matrices as follows:

$$\begin{aligned} \mathbf{U}_x(u) &= \begin{bmatrix} 1 & 0 & 0 \\ 0 & \cos(u) & -\sin(u) \\ 0 & \sin(u) & \cos(u) \end{bmatrix} \\ \mathbf{U}_y(v) &= \begin{bmatrix} \cos(v) & 0 & \sin(v) \\ 0 & 1 & 0 \\ -\sin(v) & 0 & \cos(v) \end{bmatrix} \\ \mathbf{U}_z(w) &= \begin{bmatrix} \cos(w) & -\sin(w) & 0 \\ \sin(w) & \cos(w) & 0 \\ 0 & 0 & 1 \end{bmatrix} . \end{aligned} \tag{1}$$

The yaw and roll angles are in the range - π to + π , while the pitch angle varies between -$\pi/2$ and +$\pi/2$. The displacement vector $\mathbf{R}_{cm}$ is defined via the fractional coordinates associated with the surface unit cell, with two fractional coordinates varied between 0 and 1.

II. ADSORPTION RESULTS BASED ON THE BEST NEUTRAL Mo_6 CLUSTER

Figure S1 presents the nine most energetically favorable atomic configurations (within 1.0 eV of the absolute minimum) for the Mo_6 cluster adsorbed on graphene and whose initial geometry

corresponded to that in the gas phase assuming its neutrality. The energies and the multiplicities of the systems are shown in Table SII. The spin densities of the relevant clusters are shown in Fig. S2.

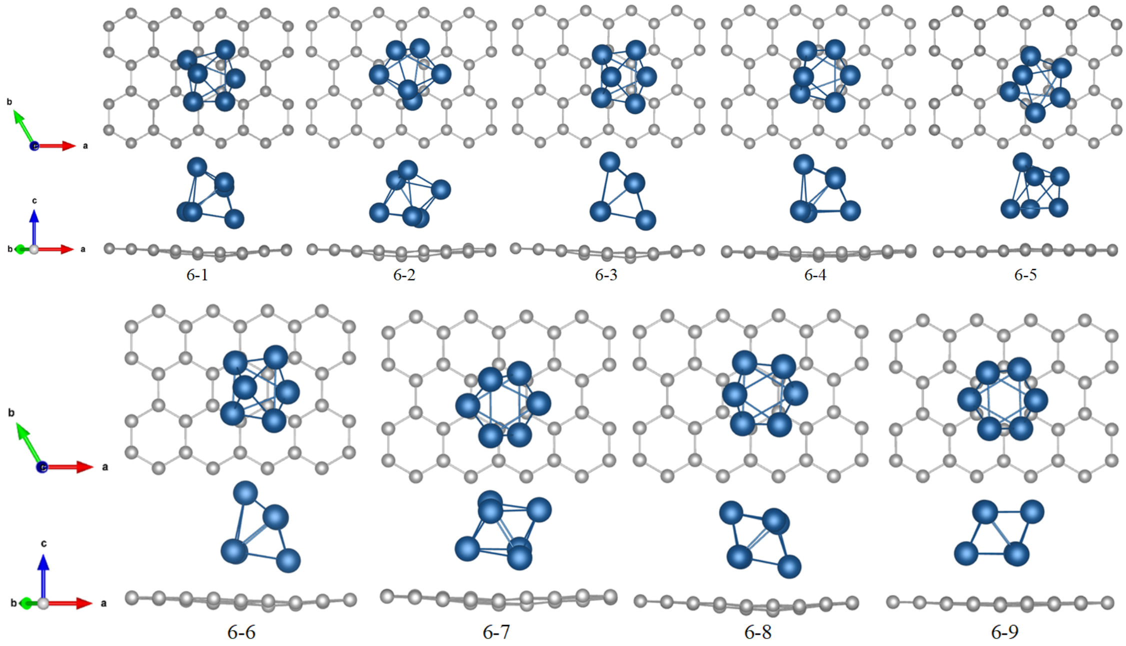

FIG. S1: Minimum energy systems (1-9) of 6 Mo atoms adsorbed on graphene.

TABLE SII: The adsorption energy E (in eV) and spin multiplicity M of 9 lowest energy structures of Mo_6 clusters on graphene found.

	6-1	6-2	6-3	6-4	6-5	6-6	6-7	6-8	6-9
adsorption energy (eV)	3.77	3.75	3.70	3.59	3.45	3.37	2.97	2.97	2.87
Spin multiplicity	1	1	3	1	3	5	7	7	7

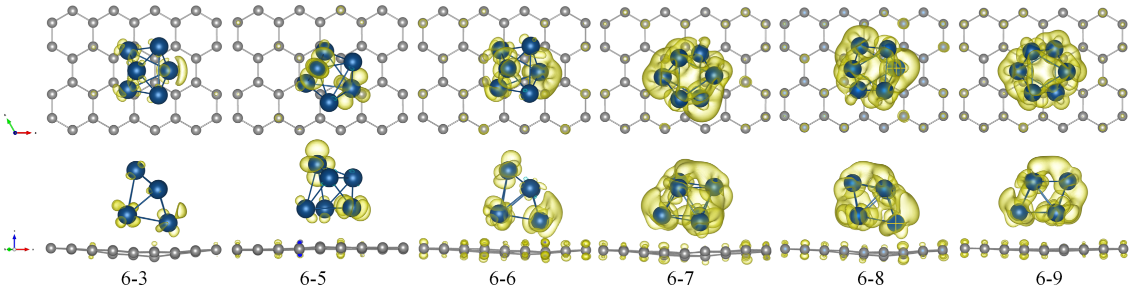

FIG. S2: The spin densities of 6-atom Mo clusters adsorbed on graphene.

III. SPIN DENSITY RESULTS BASED ON THE BEST CHARGED MO_6 CLUSTER

The spin density for selected clusters on graphene obtained assuming, as the cluster's initial geometry, its geometry with the single positive charge, is shown in Fig. S3.

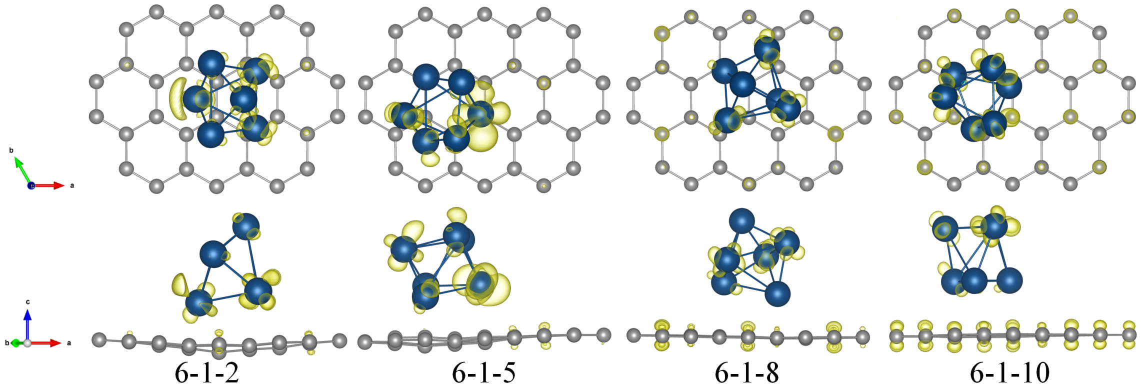

FIG. S3: Top (above) and side (below) views of the spin density (at ± 0.005 Bohr^{-3}) for selected Mo_6 clusters on graphene that demonstrate non-zero spin density ($M = 3$).

IV. THE FULL PATH FOR NEB CALCULATION

In Figures S4 and S5, we display the different geometries employed in the Nudge Elastic Band (NEB) calculations. Figure S4 shows the seven images used to compute the path, while Figure S5 displays the ten images computed for the second case as discussed in section **CO molecule absorbed on clusters on graphene**.

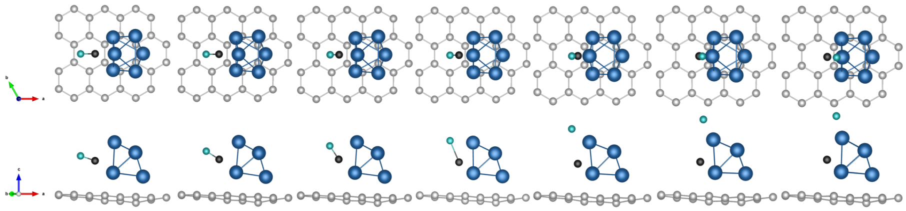

FIG. S4: All geometries along the first minimum energy path corresponding to the dissociation of the CO molecule on the Mo_6 cluster on graphene with the O atom (green) placed on top of the cluster in the final state.

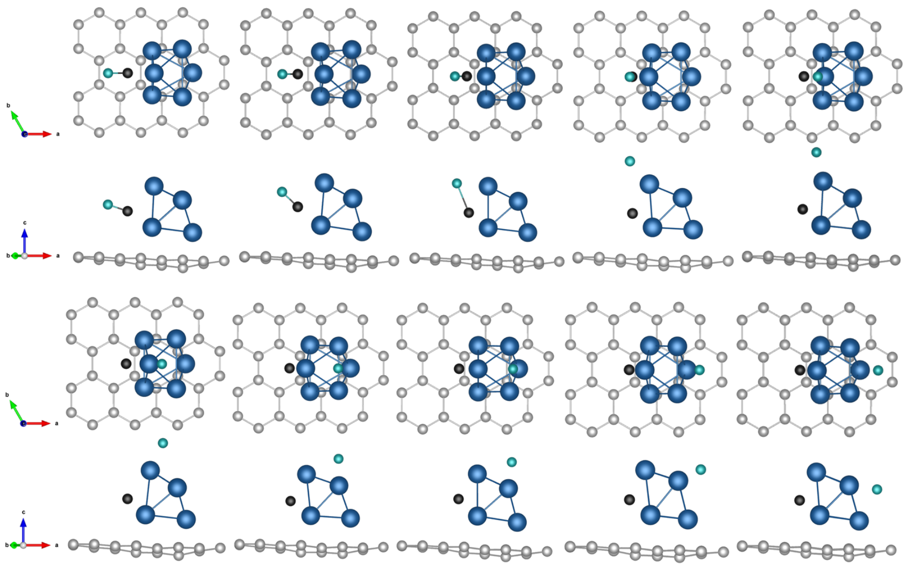

FIG. S5: All geometries along the second minimum energy path corresponding to the dissociation of the CO molecule on the Mo_6 cluster on graphene with the O atom (green) placed on the other side of the cluster.
